# APPLYING THE AGE-FRIENDLY 4MS FRAMEWORK TO IDENTIFY NEEDS OF INDIVIDUALS ENROLLED IN CDSMP

**DOI:** 10.1093/geroni/igae098.3006

**Published:** 2024-12-31

**Authors:** Abby Teply, Christine McKibbin, Joshua Clapp, Stacy Carling, Sabine Schenck, Catherine Carrico, Barbara Dabrowski, Elizabeth Punke

**Affiliations:** University of Wyoming, Laramie, Wyoming, United States; University of Wyoming, Laramie, Wyoming, United States; University of Wyoming, Laramie, Wyoming, United States; University of Wyoming, Laramie, Wyoming, United States; University of Wyoming, Laramie, Wyoming, United States; University of Wyoming, Laramie, Wyoming, United States; University of Wyoming, Laramie, Wyoming, United States; University of Wyoming, Laramie, Wyoming, United States

## Abstract

The Age-Friendly framework is an evidence-based approach to guide health care with older adults that emphasizes 4Ms: “What Matters,” “Medication,” “Mentation,” and “Mobility.” The Self-Management Resource Center’s evidence-based Chronic Disease Self-Management Program (CDSMP) aligns with this framework. Therefore, this study applied the 4Ms to identify needs of participants from CDSMP. Participants (n = 147) were predominantly White (n = 139; 94.6%) females (n = 18; 80.3%) with a mean age of 75.21 (SD = 18.83). The sample included caregivers/supporters (n = 68; 45.3%) and individuals with chronic illnesses (n = 79; 53.7%). Participants completed pre-post measures consistent with the 4Ms (e.g., provider communication, medication adherence, depressive symptoms, and exercise). A latent profile analysis was conducted to extract unobserved subgroups identified by common profiles of response at baseline. Indices of model fit suggested a two-profile model was appropriate. Profile 1 (labeled “Needs Support”) was characterized by poor medication adherence and mild depressive symptoms. High medication adherence and minimal depressive symptoms, in contrast, distinguished profile 2 (labeled “Coping Well”). T-tests revealed a significant difference between profiles on frontier and remote area (FAR) ratings, t(91.999) = -2.107, p =.038. On average, those in profile 1 lived in areas with higher FAR ratings (M = 1.10, SD = 1.24), suggesting greater remoteness, compared to those in profile 2 (M =.68, SD =.99). No differences were found on age, gender, education, etc. Rural living may complicate self-management. Additional research is needed to understand the implication of profile membership for program outcomes.

